# Validation of Pediatric Acute-Onset Neuropsychiatric Syndrome (PANS)-Related Pediatric Treatment Evaluation Checklist (PTEC)

**DOI:** 10.3390/pediatric17040081

**Published:** 2025-07-28

**Authors:** Andrey Vyshedskiy, Anna Conkey, Kelly DeWeese, Frank Benno Junghanns, James B. Adams, Richard E. Frye

**Affiliations:** 1 Metropolitan College, Boston University, Boston, MA 02215, USA; 2 Neuroimmune Foundation, Saint Augustine, FL 32084, USA; anna.conkey@neuroimmune.org (A.C.); kelly@neuroimmune.org (K.D.); fbj@raumfisch.de (F.B.J.); 3 School for Engineering of Matter, Transport and Energy, Arizona State University, Tempe, AZ 85281, USA; jim.adams@asu.edu; 4 Autism Discovery and Treatment Foundation, Phoenix, AZ 85050, USA; drfrye@autismdiscovery.org

**Keywords:** PANS, PANDAS, psychological evaluations, neuropsychiatric syndrome, streptococcal infections

## Abstract

Background/Objectives: The objective of this study was to validate a new parent-reported scale for tracking Pediatric Acute-onset Neuropsychiatric Syndrome (PANS). PANS is a condition characterized by a sudden and severe onset of neuropsychiatric symptoms. To meet diagnostic criteria, an individual must present with either obsessive–compulsive disorder (OCD) or severely restricted food intake, accompanied by at least two additional cognitive, behavioral, or emotional symptoms. These may include anxiety, emotional instability, depression, irritability, aggression, oppositional behaviors, developmental or behavioral regression, a decline in academic skills such as handwriting or math, sensory abnormalities, frequent urination, and enuresis. The onset of symptoms is usually triggered by an infection or an abnormal immune/inflammatory response. Pediatric Autoimmune Neuropsychiatric Disorders Associated with Streptococcal Infections (PANDAS) is a subtype of PANS specifically linked to strep infections. Methods: We developed a 101-item PANS/PANDAS and Related Inflammatory Brain Disorders Treatment Evaluation Checklist (PTEC) designed to assess changes to a patient’s symptoms over time along 10 subscales: Behavior/Mood, OCD, Anxiety, Food intake, Tics, Cognitive/Developmental, Sensory, Other, Sleep, and Health. The psychometric quality of PTEC was tested with 225 participants. Results: The internal reliability of the PTEC was excellent (Cronbach’s alpha = 0.96). PTEC exhibited adequate test–retest reliability (*r* = 0.6) and excellent construct validity, supported by a strong correlation with the Health subscale of the Autism Treatment Evaluation Checklist (*r* = 0.8). Conclusions: We hope that PTEC will assist parents and clinicians in the monitoring and treatment of PANS. The PTEC questionnaire is freely available at neuroimmune.org/PTEC.

## 1. Introduction

*Pediatric Acute-onset Neuropsychiatric Syndrome* (PANS) is a complex disorder characterized by the sudden onset of neuropsychiatric symptoms in children [1,2,3]. These symptoms often include obsessive–compulsive behaviors, anxiety, tics, mood instability, and cognitive decline, which appear abruptly over the course of days or even hours [4,5]. PANS presents as a dramatic shift in a child’s behavior and mental health, often causing significant distress for both the affected child and their family. *Pediatric Autoimmune Neuropsychiatric Disorders Associated with Streptococcal infections* (PANDAS) is a subtype of PANS specifically linked to streptococcal infections [6].

Currently, the *Diagnostic and Statistical Manual of Mental Disorders*, 5th edition, text revision (DSM-5-TR) does not include PANS as a syndromic entity [7]. PANS can be codified using the DSM-5-TR diagnosis of “Obsessive-Compulsive and Related Disorder Due to Another Medical Condition” (code 294.8). The International Statistical Classification of Diseases and Related Health Problems (ICD-11) does not mention PANS but includes PANDAS among the category “other specified disorders involving the immune mechanism, not elsewhere classified” with the code D89.89 [8].

Although the criteria for both PANS and PANDAS were originally established to define homogeneous groups for research purposes, there is broad consensus that these conditions are not limited to childhood and can persist beyond the pediatric population. Furthermore, Stanford’s PANS Clinic, in its characterization of the first 47 consecutive PANS patients, found that only 40% of the cohort exhibited an acute onset of symptoms [5]. Importantly, patients with and without acute onset demonstrated similar clinical profiles, including comparable symptom presentations, rates of co-occurring inflammatory conditions, somatic symptoms, and incidences of violent thoughts and behaviors. Group A Streptococcus was the most frequently identified infection both at symptom onset and during flares.

The exact cause of PANS remains uncertain. It may be triggered by bacterial (Streptococcal, *M. pneumoniae*, *B. burgdorferi*, and *S. aureus*) and viral infections (Epstein–Barr, Influenza, Coxsackie, Varicella, and SARS-CoV2); immune system dysfunction [9,10]; mitochondria dysfunction [11]; or environmental factors [12], such as emotional stress or oxidative toxin exposure, that lead to brain inflammation. Due to its sudden onset and overlapping symptoms with other psychiatric and neurological conditions, diagnosing PANS can be challenging. The persistence of immunological activation after the acute phase may be responsible for maintaining a detrimental condition. Early recognition and appropriate treatment are crucial, as interventions such as antibiotics, immunomodulatory therapies, and behavioral support can help manage symptoms and improve long-term outcomes.

Monitoring PANS is crucial for ensuring early intervention, effective symptom management, and improved long-term outcomes. Since PANS symptoms can fluctuate in severity and may be triggered by infections, stress, or immune dysfunction, regular monitoring helps identify patterns and potential triggers, allowing for timely medical and therapeutic adjustments. Without proper monitoring, individuals may experience worsening symptoms, including severe anxiety, cognitive decline, or behavioral disturbances that can interfere with their daily lives and development. Additionally, tracking an individual’s progress enables clinicians to assess the effectiveness of treatments such as antibiotics, anti-inflammatory therapies, or psychiatric interventions. By closely monitoring PANS, caregivers and clinicians can provide more targeted support, prevent relapses, and improve the child’s overall quality of life.

However, tools for monitoring PANS are currently limited and do not capture the full spectrum of related symptoms. For example, the clinician-administered *Children’s Yale-Brown Obsessive Compulsive Scale* (CY-BOCS) focuses solely on obsessive–compulsive symptoms [13], while the *Yale Global Tic Severity Scale* (YGTSS) is restricted to evaluating tics [14].

While clinician-administered instruments address only isolated aspects of the disorder, parent-reported tools specifically designed for PANS are entirely lacking—despite the fact that parents are the primary observers of their child’s behavior and health status. Given the critical need for reliable instruments to track PANS symptoms in both clinical and research settings, we developed a parent-reported tool tailored specifically for this purpose.

Our development was inspired by the *Autism Treatment Evaluation Checklist* (ATEC) [15], a widely used tool for monitoring autism symptoms, employed in clinical trials and by parents to assess their children’s progress over time. Building on the ATEC model, we developed the *PANS/PANDAS and Related Inflammatory Brain Disorders Treatment Evaluation Checklist* (PTEC), a structured tool designed to monitor symptoms associated with PANS, PANDAS, and related inflammatory brain conditions (Table 1). The PTEC was created to capture the symptom profiles of individuals who meet strict diagnostic criteria for PANS/PANDAS, as well as those exhibiting overlapping inflammatory symptoms. Its validity was tested in a cohort of 225 participants and compared with the Health subscale of the ATEC.

## 2. Methods

### 2.1. Participants

Participants in this study were individuals diagnosed with PANS, PANDAS, or another inflammatory brain disorder, or those undergoing evaluation for such a diagnosis. The majority of participants were children and adolescents, with responses provided by their caregivers. An invitation to participate in this study was emailed to caregivers who had previously registered with the Neuroimmune Foundation. As a part of the current study, demographic information about age and diagnosis of participants was collected.

### 2.2. Validation

Because PANS and PANDAS are clinical diagnoses without a validated biomarker [16], identifying a reliable benchmark for validating the PTEC presents a significant challenge. After evaluating several options, we adopted the following rationale: many symptoms of PANS overlap with those observed in Autism Spectrum Disorder (ASD). Therefore, we selected the Autism Treatment Evaluation Checklist (ATEC) [15] Health subscale as a comparative measure for validation. While we acknowledge that the ATEC Health subscale does not encompass the full spectrum of PANS symptoms, it does capture a meaningful subset.

### 2.3. Test–Retest Reliability

Test–retest reliability of the PTEC was assessed by calculating the Pearson correlation coefficient between participants’ initial and follow-up scores. Given that PANS is characterized by unpredictable flares that can significantly influence symptom severity [4,5]—and therefore PTEC scores—we aimed to have responders complete the second assessment as soon as possible after the first, while still allowing enough time to reduce recall bias. An interval of approximately three days was targeted.

### 2.4. Data Collection

The 101-item PTEC together with the 77-item ATEC [15] were administered online to 225 participants currently experiencing symptoms or in remission from these conditions. Approximately three days after the initial assessment, a follow-up evaluation was completed by 106 participants.

### 2.5. Measurements

The PTEC is a new 101-item measurement tool for tracking PANS symptoms. All subscales and items were developed by expert consensus. PTEC consists of 10 subscales (Table 1): I. Behavior/Mood (20 items), II. OCD (28 items), III. Anxiety (5 items), IV. Food intake (8 items), V. Tics (2 items), VI. Cognitive/Developmental (7 items), VII. Sensory (6 items), VIII. Other (11 items), IX. Sleep (5 items), and X. Health (9 items). For each item, responders were asked how much each of the following is a problem on a Likert-type rating scale: 0 (not a problem), 1 (minor problem), 2 (moderate problem), and 3 (serious problem). Higher scores indicate more severe symptomatology, while lower scores reflect milder symptoms.

The ATEC measurement tool is designed to be completed by parents, teachers, or caretakers and has been validated in a number of studies [17,18,19,20,21,22,23]. The ATEC was designed to monitor how well the child is doing over time [15]. In addition, researchers have used the ATEC to document improvements following an intervention by comparing the baseline ATEC scores with the post-treatment ATEC scores [24,25]. ATEC consists of 4 subscales: I. Speech/Language/Communication (14 items); II. Sociability (20 items); III. Sensory/Cognitive Awareness (18 items); and IV. Health/Physical/Behavior (25 items). Only the Health/Physical/Behavior subscale was used in this study. For simplicity, it is referred to as the Health subscale. For each item, responders were asked how much each of the following is a problem on a Likert-type rating scale: 0 (not a problem), 1 (minor problem), 2 (moderate problem), and 3 (serious problem).

### 2.6. Compliance with Ethical Standards

Using the Department of Health and Human Services regulations found at 45 CFR 46.104(d) (2), the Advarra Institutional Review Board (IRB) determined that this research project is exempt from IRB oversight. Responders have consented to anonymized data analysis and publication of the results. This study was conducted in compliance with the Declaration of Helsinki [26].

## 3. Results

### 3.1. Participants

A total of 225 participants completed both the 101-item PTEC and 25-item Health subscale of the ATEC. A total of 106 participants completed the PTEC twice, with intervals ranging from 1 to 18 days (mean ≈ 3 days). The average age of participants was 15.6 ± 8.6 (range: 2 to 59 years), and 91% had a diagnosis of PANS or PANDAS or another inflammatory brain disorder (Table 2). Among diagnosed participants, 95% were diagnosed by an MD or DO (98% were diagnosed by an MD, DO, or nurse practitioner/physician assistant). Information regarding recent illness triggers is presented in Table 3.

### 3.2. Validity

PANS and PANDAS are clinical diagnoses that currently lack universally accepted objective markers [16]. Accordingly, establishing a dependable reference point for validating the PTEC is inherently difficult. After considering various alternatives, we settled on the following reasoning: many of the symptoms seen in PANS overlap with those found in ASD. As such, we chose the Health subscale of the ATEC as a comparison tool for validation. Although the ATEC Health subscale does not fully represent the entire range of PANS symptoms, it does reflect a substantial portion of them.

For each item in PTEC and ATEC, responders rated the extent to which a symptom was a problem using a Likert-type scale: 0 (not a problem), 1 (minor problem), 2 (moderate problem), and 3 (serious problem).

**Figure 1:**
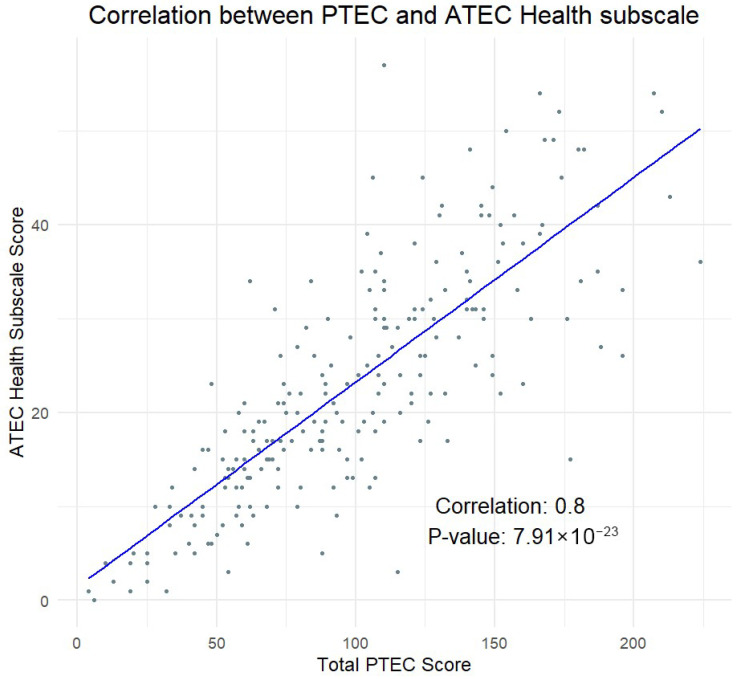
Correlation between the PTEC total score and the Health subscale of the ATEC (*r* = 0.8, *p* < 0.0001).

The PTEC showed a strong positive correlation with the Health subscale of the ATEC (*r* = 0.8, *p* < 0.0001), which testified to its convergent validity (Figure 1). This high correlation was consistent across both children (under 18 years, N = 167, *r* = 0.81, *p* < 0.0001) and adults (18 years and older, N = 58, *r* = 0.76, *p* < 0.0001).

### 3.3. Reliability

The internal consistency of the PTEC was excellent, with a Cronbach’s alpha of 0.96, indicating high overall reliability. This high Cronbach’s alpha was consistent across both children (0.97) and adults (0.96). An analysis of *‘Cronbach’s Alpha if Item Deleted’* revealed a consistent value of 0.96 across all items (Table 4), suggesting that no single item disproportionately affected the overall internal consistency of the scale.

Most PTEC items (75%) demonstrated strong item–total correlations (≥0.4). Specifically, all items in the Behavior/Mood subscale showed high item–total correlations (≥0.45), and all items in the Anxiety subscale demonstrated even stronger correlations (≥0.54). Only six items—*Daytime wetting/soiling, Bedwetting, Baby talk, Stutters, Urge to overeat, and Limited [food intake] due to choking fear*—exhibited lower item–total correlations (<0.30), indicating that these items may be less aligned with the overall construct measured by the scale.

The test–retest correlation coefficient was *r* = 0.60 (95% CI: 0.47–0.71; *p* < 0.0001), indicating moderate long-term stability of the PTEC. The test–retest correlation coefficient was consistent across both children (*r* = 0.56; 95% CI: 0.39–0.69; *p* < 0.0001) and adults (*r* = 0.72; 95% CI: 0.46–0.87; *p* < 0.0001).

## 4. Discussion

Pediatric Acute-onset Neuropsychiatric Syndrome (PANS) is characterized by the sudden onset of obsessive–compulsive symptoms and/or eating restrictions, accompanied by a broad range of neuropsychiatric manifestations [1,2,3]. A growing body of literature supports the recognition of PANS as a distinct clinical entity that warrants multidisciplinary evaluation and individualized treatment strategies.

However, diagnosing PANS remains challenging due to substantial symptom overlap with other neuropsychiatric conditions, including Tourette syndrome, early-onset schizophrenia, and various autoimmune disorders [5,27,28,29]. This diagnostic complexity highlights the importance of longitudinal monitoring, which may improve diagnostic accuracy and help identify timely treatment opportunities [30,31,32,33].

In this study, we validated a new patient-reported instrument—the 101-item PANS/PANDAS and Related Inflammatory Brain Disorders Treatment Evaluation Checklist (PTEC)—designed to track changes in a child’s symptoms over time across ten broadly defined subscales: Behavior/Mood, Obsessive–Compulsive Symptoms (OCD), Anxiety, Food Intake, Tics, Cognitive/Developmental, Sensory, Other, Sleep, and Health. These subscales group the 101 items into loosely organized categories; therefore, it was not appropriate to analyze the subscales separately.

The PTEC is not intended as a diagnostic tool but rather as an instrument for tracking and comparing symptom severity across two or more time points. It can be used by patients, parents, or physicians to assess changes before and after a treatment, during or following a flare, or to establish a baseline measure.

At present, there is no established gold standard for evaluating PANS severity, which poses a challenge in selecting an appropriate benchmark for validating the PTEC. After considering several alternatives, we adopted the following rationale: many PANS symptoms overlap with those seen in Autism Spectrum Disorder (ASD). As such, we chose the Health subscale of the Autism Treatment Evaluation Checklist (ATEC) [15] as a comparative measure. The psychometric properties of the PTEC were evaluated using data from 225 respondents. The PTEC demonstrated excellent construct validity, as evidenced by a strong correlation with the Health subscale of the ATEC (*r* = 0.80).

To evaluate the internal consistency of the PTEC, we conducted a reliability analysis across all items. The results indicated excellent internal reliability, with a Cronbach’s alpha of 0.96. Most items demonstrated strong item–total correlations (≥0.40), supporting the cohesiveness of the scale. Only six items—*Daytime wetting/soiling*, *Bedwetting*, *Baby talk*, *Stutters*, *Urge to overeat*, and *Limited [food intake] due to choking fear*—showed lower item–total correlations (<0.30), suggesting these items may be less closely aligned with the overall construct measured by the PTEC. Further research with a larger sample size is needed to determine the relevance of the six items with lower item–total correlations.

One of the most striking characteristics of PANS is the abrupt onset of symptoms, which can cause significant distress for both patients and their families [4,5]. In light of this, the test–retest reliability assessment was scheduled with a short interval of approximately three days. Despite this brief interval, several responders reported the emergence of new symptom flares, which influenced their responses. However, no participants were excluded from the analysis, as it was not possible to confirm whether all flares had been consistently reported. Even with these fluctuations, the PTEC demonstrated adequate test–retest reliability (*r* = 0.60). Future studies could ask all participants to report any new flares occurring between evaluations as part of the survey. Excluding these participants from the test–retest analysis is expected to improve the accuracy of the test–retest reliability.

One limitation of this study is the reliance on patient-reported diagnoses. The majority of participants (91%) reported a diagnosis of PANS, PANDAS, or another inflammatory brain disorder, while 11% reported that they were undiagnosed (Table 2). The 2% overlap is likely due to the checkbox format used to collect diagnostic information, suggesting that some respondents may have inadvertently selected both options. Among diagnosed participants, 95% reported that the diagnosis was made by a licensed medical professional (MD or DO) and 98% were diagnosed by an MD/DO or Nurse Practitioner/Physician’s Assistant. Most participants had unknown illness triggers (Table 3). Including 11% undiagnosed participants could dilute this study’s focus on confirmed PANS cases, potentially skewing results related to symptom severity. However, reporting average symptom severity was not among this study’s objectives. Accordingly, all participants who completed the PTEC were included in the analysis, regardless of diagnostic status.

Future research should focus on longitudinal studies to better understand the natural course of PANS, identify biomarkers of disease activity, and assess the efficacy of specific treatments. Additionally, studies into the psychosocial impact of PANS on families and the role of early intervention are warranted.

The PTEC questionnaire is freely available at neuroimmune.org/PTEC (accessed on 11 April 2025). Code availability statement: The code is available from the corresponding author upon reasonable request.

## Figures and Tables

**Table 1:** List of PTEC items. For each item, responders were asked to rate the extent to which each issue was a problem using the following scale: *not a problem*, *minor problem*, *moderate problem*, or *serious problem*.

#		Subscale/Item
		**I. Behavior/Mood**
1	1	Rages
2	2	School refusal
3	3	Uncooperative or resistant
4	4	Oppositional behavior
5	5	Disagreeable/non-compliant
6	6	Temper tantrums
7	7	Shouts or screams
8	8	Insensitive to others’ feelings
9	9	Inappropriate laughing/crying
10	10	Disinhibited/impulsive
11	11	Avoids contact with others
12	12	Harms self
13	13	Irritable/agitated
14	14	Destructive to property
15	15	Verbal aggression
16	16	Physical aggression
17	17	Unhappy/crying
18	18	Depressed mood
19	19	No motivation/joy
20	20	Mood swings
		**II. OCD**
21	1	Unwanted sexual thoughts
22	2	Violent/horrific intrusive images
23	3	Non-violent intrusive thoughts
24	4	Obsession/fear of germs
25	5	Washing/cleaning compulsions
26	6	Fear chemicals/solvents/contaminates
27	7	Fears harm will come to self
28	8	Fears hurting self/others
29	9	Fears will be responsible for aggressive/illegal behavior
30	10	Fear of blurting obscenities
31	11	Perfectionism
32	12	Checking or repeating obsessions/rituals
33	13	Counting rituals
34	14	Destructive to property
35	15	Confessing
36	16	Reassurance seeking
37	17	Obsessive speech
38	18	Repetitive speech
39	19	Rigid routines
40	20	Hoarding rituals
41	21	Magical thoughts/superstitious obsessions
42	22	Lucky/unlucky words, numbers, colors
43	23	Requires symmetry
44	24	Arranging obsessions
45	25	Obsession with order/location of objects
46	26	Need to tap or touch
47	27	Overly concerned with health
48	28	Requires another person to participate in OCD
		**III. Anxiety**
49	1	Separation anxiety
50	2	Irrational or amplified fears
51	3	Avoids leaving home
52	4	Anxious about being around people
53	5	General/other anxiety
		**IV. Food intake**
54	1	Food refusal/anorexia
55	2	Limited fluid intake
56	3	Picky eating
57	4	Negative body image
58	5	Limited due to choking fear
59	6	Limited due to fear of vomiting
60	7	Limited due to contamination fear
61	8	Urge to overeat
		**V. Tics**
62	1	Vocal tics
63	2	Motor tics
		**VI. Cognitive/Developmental**
64	1	Memory issues
65	2	Brain fog
66	3	Stutters
67	4	Baby talk
68	5	Gross motor regression (tripping/coordination issues)
69	6	Fine motor: difficulty with/regression handwriting/art
70	7	Difficulty with/regression math skills
		**VII. Sensory**
71	1	Sound-sensitive
72	2	Light-sensitive
73	3	Sensitive to smells
74	4	Sensitive to textures
75	5	Other sensory sensitivities
76	6	Amplified sensory seeking
		**VIII. Other**
77	1	Hallucinations
78	2	Delusions
79	3	Paranoia
80	4	Suicidal ideation
81	5	Homicidal ideation
82	6	Attention issues
83	7	Hyperactivity
84	8	Daytime urinary frequency
85	9	Daytime wetting/soiling
86	10	Lacks friends
87	11	Dilated pupils
		**IX. Sleep**
88	1	Nightmares
89	2	Night terrors
90	3	Problems falling asleep
91	4	Problems staying asleep
92	5	Bedwetting
		**X. Health**
93	1	Constipation
94	2	Diarrhea
95	3	Acute infection
96	4	Fatigued
97	5	Lethargic
98	6	Pain in stomach
99	7	Pain in head
100	8	Pain in joints
101	9	Pain other

**Table 2:** Participants’ demographics.

	Number of Participants	Percent of Total	Age, Mean (SD)
PANS, PANDAS, or Other inflammatory brain disorder	204	91	15.6 (8.0)
PANS	135	59	16.0 (8.5)
PANDAS	91	40	14.1 (6.2)
Other inflammatory brain disorder	50	22	18.7 (7.3)
Undiagnosed	25	11	16.1 (13.0)

**Table 3:** Recent illness trigger.

	Number of Participants	Percent of Total	Age, Mean (SD)
Group A Strep exposure without infection	23	10	14.8 (10.8)
Group A Strep confirmed infection	48	21	13.8 (7.3)
COVID-19	36	16	17.8 (10.1)
Influenza	22	10	12.7 (5.3)
Viral infection (other)	60	27	14.7 (8.3)
Bacterial infection (other)	53	24	16.0 (8.9)
Allergen exposure	21	9	18.5 (10.7)
Unknown	87	37	16.5 (8.5)

**Table 4:** Item–total correlations.

	#		Item	Item–Total Correlation	Alpha If Item Deleted
**I. Behavior/Mood**	1	1	Rages	0.56	0.96
	2	2	School refusal	0.58	0.96
	3	3	Uncooperative or resistant	0.59	0.96
	4	4	Oppositional behavior	0.59	0.96
	5	5	Disagreeable/non-compliant	0.59	0.96
	6	6	Temper tantrums	0.56	0.96
	7	7	Shouts or screams	0.58	0.96
	8	8	Insensitive to others’ feelings	0.45	0.96
	9	9	Inappropriate laughing/crying	0.53	0.96
	10	10	Disinhibited/impulsive	0.49	0.96
	11	11	Avoids contact with others	0.51	0.96
	12	12	Harms self	0.42	0.96
	13	13	Irritable/agitated	0.63	0.96
	14	14	Destructive to property	0.45	0.96
	15	15	Verbal aggression	0.55	0.96
	16	16	Physical aggression	0.55	0.96
	17	17	Unhappy/crying	0.63	0.96
	18	18	Depressed mood	0.62	0.96
	19	19	No motivation/joy	0.59	0.96
	20	20	Mood swings	0.66	0.96
**II. OCD**	21	1	Unwanted sexual thoughts	0.4	0.96
	22	2	Violent/horrific intrusive images	0.5	0.96
	23	3	Non-violent intrusive thoughts	0.52	0.96
	24	4	Obsession/fear of germs	0.39	0.96
	25	5	Washing/cleaning compulsions	0.34	0.96
	26	6	Fear chemicals/solvents/contaminates	0.36	0.96
	27	7	Fears harm will come to self	0.45	0.96
	28	8	Fears hurting self/others	0.43	0.96
	29	9	Fears will be responsible for aggressive/illegal behavior	0.45	0.96
	30	10	Fear of blurting obscenities	0.42	0.96
	31	11	Perfectionism	0.41	0.96
	32	12	Checking or repeating obsessions/rituals	0.47	0.96
	33	13	Counting rituals	0.37	0.96
	34	14	Destructive to property	0.43	0.96
	35	15	Confessing	0.37	0.96
	36	16	Reassurance seeking	0.49	0.96
	37	17	Obsessive speech	0.56	0.96
	38	18	Repetitive speech	0.48	0.96
	39	19	Rigid routines	0.53	0.96
	40	20	Hoarding rituals	0.43	0.96
	41	21	Magical thoughts/superstitious obsessions	0.39	0.96
	42	22	Lucky/unlucky words, numbers, colors	0.33	0.96
	43	23	Requires symmetry	0.4	0.96
	44	24	Arranging obsessions	0.37	0.96
	45	25	Obsession with order/location of objects	0.47	0.96
	46	26	Need to tap or touch	0.35	0.96
	47	27	Overly concerned with health	0.36	0.96
	48	28	Requires another person to participate in OCD	0.45	0.96
**III. Anxiety**	49	1	Separation anxiety	0.61	0.96
	50	2	Irrational or amplified fears	0.68	0.96
	51	3	Avoids leaving home	0.54	0.96
	52	4	Anxious about being around people	0.59	0.96
	53	5	General/other anxiety	0.69	0.96
**IV. Food intake**	54	1	Food refusal/anorexia	0.5	0.96
	55	2	Limited fluid intake	0.38	0.96
	56	3	Picky eating	0.45	0.96
	57	4	Negative body image	0.33	0.96
	58	5	Limited due to choking fear	0.24	0.96
	59	6	Limited due to fear of vomiting	0.42	0.96
	60	7	Limited due to contamination fear	0.41	0.96
	61	8	Urge to overeat	0.19	0.96
**V. Tics**	62	1	Vocal tics	0.34	0.96
	63	2	Motor tics	0.39	0.96
**VI. Cognitive/Developmental**	64	1	Memory issues	0.45	0.96
	65	2	Brain fog	0.56	0.96
	66	3	Stutters	0.26	0.96
	67	4	Baby talk	0.28	0.96
	68	5	Gross motor regression (tripping/coordination issues)	0.42	0.96
	69	6	Fine motor: difficulty with/regression handwriting/art	0.48	0.96
	70	7	Difficulty with/regression math skills	0.51	0.96
**VII. Sensory**	71	1	Sound-sensitive	0.56	0.96
	72	2	Light-sensitive	0.48	0.96
	73	3	Sensitive to smells	0.51	0.96
	74	4	Sensitive to textures	0.49	0.96
	75	5	Other sensory sensitivities	0.41	0.96
	76	6	Amplified sensory seeking	0.45	0.96
**VIII. Other**	77	1	Hallucinations	0.42	0.96
	78	2	Delusions	0.48	0.96
	79	3	Paranoia	0.58	0.96
	80	4	Suicidal ideation	0.35	0.96
	81	5	Homicidal ideation	0.33	0.96
	82	6	Attention issues	0.58	0.96
	83	7	Hyperactivity	0.51	0.96
	84	8	Daytime urinary frequency	0.46	0.96
	85	9	Daytime wetting/soiling	0.25	0.96
	86	10	Lacks friends	0.45	0.96
	87	11	Dilated pupils	0.54	0.96
**IX. Sleep**	88	1	Nightmares	0.43	0.96
	89	2	Night terrors	0.42	0.96
	90	3	Problems falling asleep	0.37	0.96
	91	4	Problems staying asleep	0.4	0.96
	92	5	Bedwetting	0.2	0.96
**X. Health**	93	1	Constipation	0.36	0.96
	94	2	Diarrhea	0.39	0.96
	95	3	Acute infection	0.47	0.96
	96	4	Fatigued	0.44	0.96
	97	5	Lethargic	0.45	0.96
	98	6	Pain in stomach	0.34	0.96
	99	7	Pain in head	0.41	0.96
	100	8	Pain in joints	0.48	0.96
	101	9	Pain other	0.43	0.96

## Data Availability

De-identified raw data from this manuscript are available from the corresponding author upon reasonable request.
